# “It’s too hard” – the management of latent TB in under-served populations in the UK: a qualitative study

**DOI:** 10.1186/s12913-022-08855-w

**Published:** 2022-12-01

**Authors:** Adam Thorburn Gray, Julian Surey, Hanif Esmail, Alistair Story, Magdalena Harris

**Affiliations:** 1 Find and Treat, University College London Hospitals, London, UK; 2 Hospital for Tropical Diseases, University College London Hospitals, London, UK; 3 Institute for Global Health, University College London, London, UK; 4 MRC Clinical Trials Unit, University College London, London, UK; 5 Collaborative Centre for Inclusion Health, University College London, London, UK; 6 Department of Public Health, Environments and Society, London School of Hygiene and Tropical Medicine, London, UK

**Keywords:** Latent tuberculosis infection, Under-served populations, Policy

## Abstract

**Background:**

UK national guidance recommends systematic screening for latent tuberculosis infection (LTBI) in under-served populations, including people experiencing homelessness and people who use drugs. This is not routinely implemented in the UK, and the reasons for this policy-practice mismatch remain underexplored.

**Methods:**

Semi-structured qualitative interviews were conducted with 19 healthcare professionals from across the UK. Participants were recruited using purposive sampling and snowballing, identifying individuals with excellent knowledge of their regions practice and policy of LTBI management. The interviews were conducted online, and were audio recorded, with transcripts thematically analysed using a two-stage inductive coding process to explore perceived barriers and enablers to LTBI screening.

**Results:**

Most participants had previous experience managing LTBI in under-served populations, but none were conducting systematic screening as per national guidance. We identified service provision challenges and low prioritisation of LTBI as the key explanatory themes driving this policy-practice mismatch. Lack of resource, and the complexity of clinical decision making were two key service level barriers. System and service inertia, and lack of cost effectiveness evidence led to LTBI being deprioritised. Service integration and promotion of WHO targets for TB elimination were highlighted as potential solutions.

**Conclusion:**

Integrating LTBI testing and treatment with existing health services for under-served populations could improve feasibility and efficacy. Promotion of UK TB elimination goals and generation of regional evidence to support commissioning for LTBI care is vital. Without such a multi-pronged approach inertia is likely to persist and the zeitgeist will remain: “it’s too hard”.

**Supplementary Information:**

The online version contains supplementary material available at 10.1186/s12913-022-08855-w.

## Background

Latent tuberculosis infection (LTBI) is defined as the evidence of immune sensitisation to tuberculosis (TB), suggesting infection with *Mycobacterium tuberculosis*, but with no clinical features of active disease. It is estimated that a quarter of the world has LTBI, with approximately 5–10% going on to develop active TB [1]. Treatment options are available to reduce the risk of progression, which therefore have an important role in the goal to eliminate TB. Systematic screening of individuals at high risk for acquiring infection (recent contacts) or progression to disease (immunocompromised) is routine throughout the UK and other high income countries, with screening of migrants from high burden countries also conducted in some places, an approach found to be cost-effective [2].

The National Institute for Health and Care Excellence (NICE) guidance [3] in the UK recommends that under-served populations, including people experiencing homelessness and people who use drugs [4], receive systematic screening for LTBI as part of a broader approach to improve health outcomes. However, despite epidemiological support showing these groups to have consistently higher rates of TB infection (16.5% in London [5]), disease, and mortality when compared to the general population [6], screening is not conducted in the UK. This disproportionate effect of TB on under-served groups is, in fact, found globally and is independent of country-level burden of TB [1, 5, 7–11]—World Health Organisation (WHO) guidance, therefore, also recommends systematic screening in these key populations globally [1].

The UK has a low incidence of TB and is targeting disease elimination [12], an activity co-ordinated by regional TB Control Boards (TBCB). These are teams comprising public health and clinical healthcare professionals, although the COVID-19 pandemic has had a serious impact on their function. Routine reporting from the TBCBs highlight geographical and social heterogeneity in the prevalence of LTBI in the UK (Table 1) [6], posing challenges for a uniform approach.

**Table 1:** Regional epidemiology for TB in England (UKHSA 2020 Report)

Region	Number of TB notifications	Rate per 100 000 (95% CI)	> = 1 SRF^a^
London	1655	18.5 (17.6 – 19.4)	13.1%
West Midlands	580	9.8 (9.0 – 10.6)	17.7%
North West	524	7.1 (6.5 – 7.8)	10.8%
South East	506	5.7 (5.2 – 6.2)	11.9%
East of England	413	6.3 (5.8—7.0)	12.9%
Yorkshire & Humber	356	6.5 (5.8 – 7.2)	16.0%
East Midlands	310	6.4 (5.7 – 7.1)	14.7%
South West	234	4.2 (3.6 – 4.7)	17.6%
North East	84	3.1 (2.5 – 3.9)	16.4%
**England**	4725	8.4 (8.2 – 8.6)	13.9%

^a^ Social Risk Factors: current or history of illicit drug use, current heavy alcohol use, current or history of homelessness, current or history of imprisonment

In recent years in the UK there has been an increased recognition, and improved management of active TB in under-served populations. Published in 2020, *Tackling TB in under-served populations* outlined “exemplars of good practice” including housing provision, cross-sector collaboration, and forming integrated care plans with other services [10]. However, care models for active and latent TB are different and translating these “exemplars of good practice” will require an understanding of LTBI-specific challenges. Previous research in this area has focused on barriers within migrant communities [13], or in high burden settings [14], and are not likely to be directly applicable to the under-served populations in the UK and other high-income countries.

In this paper we report findings from qualitative data generated with TB providers across the UK, exploring regional experiences with LTBI management in under-served populations. An understanding of the barriers and enablers to delivering national guidance – the systematic screening of LTBI in under-served groups – is crucial to enable change in policy and practice.

## Methods

### Study design and participants

This qualitative study aimed to explore regional variation in policy and practice in LTBI management for under-served populations, with a focus on understanding barriers to enacting national guidance, and to inform future pilot studies. It has been carried out and reported in line with COREQ guidelines for qualitative research [15].

LTBI management in the UK is often nurse-led, provided in secondary care settings (e.g. hospital-based) alongside community staff (e.g. TB nurse specialists). Study participants were sampled from healthcare workers identified through discussion with national leads, and snowballing, as having an extensive working knowledge of their local practice and policy for the management of LTBI in under-served populations. We aimed to recruit a heterogeneous group, including clinicians (respiratory, infectious diseases, public health) and specialist nurses from across the UK. Potential participants were contacted by email through existing clinical networks, given information about the study, and informed, written consent was obtained by asking participants to return a signed, completed form.

**Fig. 1:**
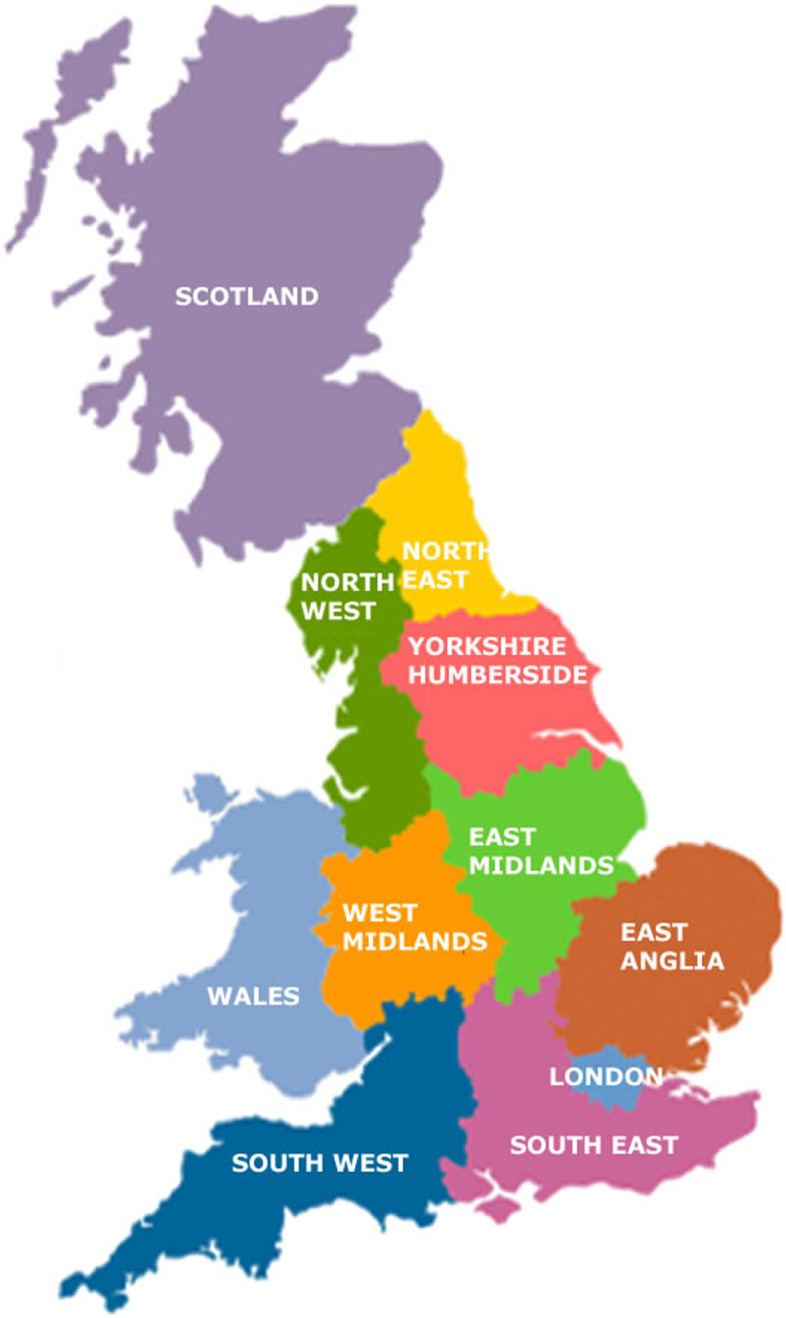
Map of TB control administrative regions

Participants were recruited from a wide geographical area including Wales, Scotland and all nine administrative regions in England (Fig. 1). Although many held responsibility over large areas, the healthcare structure for TB management in the UK meant they were not considered to necessarily ‘represent’ their entire region, as practice and policy were expected to vary within regions.

### Data collection

Data was collected using semi-structured interviews conducted online using video-call technology, between February and April 2022 by a single researcher (AG), a male clinician with experience in qualitative research and the management of LTBI. The interviews took between 30–70 min, were audio-recorded and transcribed, with the transcriptions anonymised for analysis alongside field notes taken at the time of interview. The interviews were structured around a topic guide, developed through discussions with experts in the field (Supplementary Material). Topic guide domains comprised: past and present regional practice for the management of LTBI in under-served populations and perceptions of barriers or enablers to LTBI implementation. Given the known deficit in systematic screening of LTBI among under-served groups, the interviews sought to quickly establish current practice, before further exploring reasons for mismatch between practice and policy.

Recruitment continued in parallel with analysis until the researchers felt no new information was generated, and that an interview had been conducted with at least one participant from each region. During the process small, iterative changes were made to the topic guide, with additional questions introduced to further explore topics of importance arising in the first few interviews.

### Data analysis

Transcripts were not returned to participants for verification prior to analysis. Transcripts were read through independently by two researchers (AG, JS), to familiarise themselves with the data, and then to conduct initial coding. Each transcript was reviewed and coded several times, at different stages throughout data collection, to ensure coding was comprehensive. These codes were then developed into themes independently by the same two researchers. These themes were reviewed and refined, in line with the inductive thematic analysis framework outlined by Braun & Clarke [16], through a series of group discussions (AG, JS, MH, HE, AS). This process continued iteratively until the themes felt comprehensive and made sense intuitively.

## Results

### Participant demographics

Thirty four individuals were contacted, 16 from the initial list of potential participants, and 18 through snowballing. 17/34 (50%) participants agreed to and completed interviews, two of whom invited a second person from their team to join the same interview, resulting in 19 participants in total (Table 2). 12 contacted individuals did not respond, three declined to participate but offered details of another colleague, and two agreed, but a suitable time was not found. Table 2: Participant characteristics.

**Table 2:** Participant characteristics

Characteristic	Number (%)
**Role**	
Clinician (non-public health)	13 (68%)
—Consultant in Respiratory Medicine	**- **7/13 [6 = clinical lead]
—Infectious Diseases Consultant	**- **6/13 [3 = clinical lead]
Public Health Clinician	3 (16%)
TB Clinical Nurse Specialist	3 (16%)
**Gender**	
- Male	121212121212(63%)
- Female	7 (37%)

Many clinicians (9/13) were clinical leads for TB at their NHS-trust – a role that encompasses regional responsibilities for TB prevention and care – with the remainder being experienced TB clinicians with longitudinal knowledge of their departmental practice. TB Clinical Nurse Specialists (CNS) in the UK are often responsible for management of people with LTBI, and so whilst they were not clinical leads for their departments, they also had excellent knowledge and experience on this topic, including participating in their regional TB Control Board (TBCB) meetings. Public Health clinicians generally had no day-to-day involvement in patient care but were usually responsible for regional TBCB strategy, and the co-ordination of TB outbreak management.

### Regional LTBI management

All participants reported routine, systematic screening for contacts of people with active TB, new healthcare workers, and patients starting on biologic agents, with some screening new migrants if commissioned. Some participants reported LTBI screening in other groups including patients receiving dialysis, solid-organ transplants, and prior to chemotherapy. No participants reported systematic screening of any under-served population, although many teams did have previous experience with research- or charity-funded projects, run as pilot studies in homeless hostels, prisons, and drug and alcohol services. None of these pilots were on-going, either due to lack of funding or COVID-19 related pressures. We found no published literature describing these pilots.

All participants reported using 3HR (three months of daily rifampicin and isoniazid) as the first line treatment option, using 6H (six months of daily isoniazid) as a second option. Experience with 4R (four months of daily rifampicin) or 3HP (three months of weekly rifapentine and isoniazid), regimens potentially well suited to under-served populations and those requiring observed treatment, was limited to sporadic use in one region. No services used peer support in testing or treatment.

### Why is there a policy-practice mismatch?

The interviews explored barriers and enablers to the systematic application of NICE guidance to investigate the mismatch with current practice, and we present results against two over-arching themes: service provision challenges and prioritisation.

### Service provision challenges “It’s hard!”

*“I think it has been neglected because it was felt to be too hard”* (#5).

An overarching narrative across the data was that provision of an LTBI service to under-served populations was just “too hard”, with analysis of accounts generating three underpinning themes: lack of resource, the inherent complexity of LTBI diagnosis and treatment in these groups, and potential solutions involving integrating with other services.

#### Lack of resource

Most participants cited lack of resource as a major barrier. There was general recognition that providing LTBI care to under-served populations required significantly more resource when compared to the general population, largely due to the increased nursing time required to ‘chase’ people who did not attend appointments: “*TB nurse time is the sticking point*” (#1). Comparisons were made to managing active TB in these key populations, which typically took up a lot of nursing time, but was appropriately funded and considered to offer more important patient and public health outcomes (e.g. reducing risk of death, risk of transmission). A deficit of specific resources, such as x-ray machines in prisons, was also mentioned as a barrier to screening provision.

When asked to expand on why management of TB in under-served populations was so labour-intensive, participants accounts orientated toward describing under-served populations as ‘hard-to-reach’: “*we don’t have the capacity to ask TB nurses to go chase them*” (#7); “*they are, by their very nature, slightly chaotic*” (#2). Some, however, offered the idea that it might be the TB services that were ‘hard-to-reach’ for these clients:

*“and actually physically getting them to come up to the clinic. A lot of issues with high DNA (did not attend) rates one has to be very creative about thinking about how you can offer services that are going to be accessible to that group”* (#10)

The study was conducted against a backdrop of the Covid-19 pandemic. This was noted by most participants as a ‘resource distractor’ from provision of TB care. The pandemic disproportionately required the services of public health, infectious disease, and respiratory healthcare professionals, with low priority tasks including LTBI often the first service to be paused: “*like everything else [LTBI management] has been vastly disrupted by the COVID pandemic, and we’re just sort of waiting, you know*…” (#12).

A lack of funding, linked to an absence of local or national commissioning for LTBI management in this group, was identified by all participants as a key barrier to service provision:

*“it’s always the commissioning policy side of things that stops us, or makes it hard for us to do what we think is very obvious that needs to be done”* (#6)

Although several services had previous pilot experience providing LTBI to under-served populations, no one reported generating data to evidence an argument for commissioning. Whilst some pilots had been unsuccessful due to challenges linking people into care, others had reportedly worked well. This tension between attributing a service deficit to limited funding and not generating the evidence to support increased funding is further explored in theme two: prioritisation.

#### Complex decision making

Even if well resourced, participants still had concerns over the ability of a service to easily and effectively identify individuals who would gain benefit from LTBI treatment. In addition to the well-recognised limitations of an IGRA (interferon-gamma release assay) in predicting risk of progression to active TB [17], some participants reported an anxiety regarding the overlap of symptoms between active TB and drug use/withdrawal (e.g. cough, sweating) and the increased likelihood of abnormal radiology due to increased rates of smoked drug use (e.g. crack lung) and cigarette smoking in this population: “*they’ve got dodgy chest x-rays, they all have a cough, so they’re going to need a bit of thinking about before you whack them on some latent treatment*” (#16). The perceived reduced specificity of these tools (e.g. symptom screening, radiology) made decision making more difficult. There were no solutions offered by participants for these concerns; we as authors are not aware of an evidence base to support or refute these hypotheses.

Decision making regarding treatment options was also complex, with participants raising concerns about risks of treatment including specific drug-drug-interactions more common in under-served populations (e.g. rifampicin-opiate) and the increased risk of drug-induced hepatotoxicity in people drinking excess alcohol:

“*getting the IGRA done, and then come back and talk about treatment and give treatment in a safe way... LFT check is difficult... Drinking heavily, concerns about giving them rifampicin and … it’s tough! I don’t have an answer for that*…” (#17)

While there was acknowledgement that “*these regimens don’t really lend themselves to being given easily*” (#5), the complexities of the patient population were also perceived as a barrier to care provision – in relation to maintaining communication (consistent mobile phone access, for example) but also due to concerns about ‘chaotic’ use of illicit drugs and interactions with drug treatment medications: “*pretty reluctant to embark on a treatment of something that we couldn’t monitor and we couldn’t work out whether it was causing problems with, you know, methadone”* (#1).

Here lies a tension, regarding assessing and communicating the risks and benefits of a potentially toxic but also preventative treatment: “*persuading the client that it’s important is difficult” (#17).* While this is an issue for all patient groups, it can be particularly challenging when people from underserved populations are faced with more immediate priorities:

“…*whether the individuals themselves wish to take action in a setting where they’re not really that interested in preventative therapy… … maybe that’s a value judgement… probably much more interested in getting food and etc*” (#5)

#### Integration with other services

Service integration was cited by many participants as a potential solution to the barriers discussed above. Embedding LTBI provision within other services which work effectively with under-served populations could reduce resource- and complexity-related constraints to care. Given the multiple issues faced by key populations – physical, psychological, social—a holistic approach was recommended by many:

“*These groups, they are not your traditional patients, and I guess a more holistic approach needs to be adopted, and I think that change in mindset takes a bit of time*” (#2).

Participants reflected on the success of prior pilots in which additional supports were provided or LTBI screening was embedded in with other services. Housing and benefit support, for example, was cited as a way in which to effectively engage people with TB screening and treatment uptake:

“*We also looked at wider issues, you know some of them have drug and alcohol [issues], or wound management ... housing benefits ... So, it was a major success*” (#5)

“*We've had lots of successes, I think with some patients treated for active TB where the TB nurses have really provided quite a holistic service, have supported patients with accommodation in hostels or you know all sorts of issues, not necessarily infection control ones*” (#10)

A ‘one-stop-shop’ was seen to provide a better service for the patient, be more feasible, cost efficient, and more likely to attract funding and commissioning for TB related care:

“*if you want to sell anything to local stakeholders just with TB, you will not find any buyers because it's small numbers, you will have to sell it as part of a package for these vulnerable populations*” (#7)

Participants highlighted several potential services with which LTBI management could be integrated, including hepatitis C (HCV), specialist primary care, and drug & alcohol services. HCV treatment teams were widely lauded as having excellent engagement with underserved populations, good funding, and strong leadership. Specialist primary care settings for people experiencing homelessness were reported to benefit from being in the right place, usually the city centre “*they can walk to clinic if they want, it’s not logistically difficult*” (#4), which facilitates service engagement, providing opportunity for integrated disease management.

“*The Hep C team… they've tagged onto our new setup for migrant screening, but perhaps we can take that the other way and try and get into [their setup]*” (#14)

It was also noted by several participants that integrating into services that had reasonably fixed relationships with patients (e.g. opioid substitution therapy services, prisons), could be helpful enabling ongoing engagement and reducing loss to follow-up among key populations: “*I guess in terms of incarcerated individuals, I guess the barriers to that are going to be smaller, because you’ve obviously got, as it were, a captive audience*” (#2).

### Prioritisation

These solutions, including recourse to the term “captive audience” arise in a context whereby LTBI is rarely prioritised and often overlooked in public health policy, commissioning, and clinical practice. While active TB case management was accorded greater attention than LTBI, many participants felt that LTBI was also deprioritised in relation to non-communicable disease management (cancer and cardiovascular disease) in general populations. As noted above, de-prioritisation and lack of funding was linked to a dearth of evidence to influence commissioning priorities: “… *it would be nice to know what the actual cost-effectiveness of what you were doing was*…” (#15). While previous pilots were talked of with some pride, there was no reference to evaluation of this work, or evidence generation to promote sustainable implementation: “*I have not seen anywhere the economic evaluation*” (#9). This appears counterintuitive when considering the remit of service provision pilots yet might reflect the perceived clinical inertia and lack of leadership, as reported below.

#### Inertia and drive

Participants spoke of a lack of ‘top-down drive’ and support for LTBI management, generally from the local TBCBs. Even prior to Covid-19 related disruption, many cited the functionality of these groups as variable, with limited interest outside of outbreaks and limited communication with the associated clinical teams: *“we really only communicate if there’s an outbreak…” (#1)*; *“there’s a traditional disconnect between public health and the clinicians” (#3)*. It was acknowledged that TBCBs were faced with significant challenges due to operating over a large geographical area with a mix of low-prevalence rural and higher-prevalence urban areas: “*so the control board I think is a bit dysfunctional because what it’s trying to cover is so disparate*” (#16). Nonetheless, it was felt by many that TBCBs lacked the strong leadership and organisation required to implement real change:

“*in my five years here…, I have never seen anything from our control board to give direction as to what we should be doing in terms of our local strategy”* (#15)*.*

There also appeared to be significant inertia from clinicians, who were often sceptical regarding whether screening was worth doing, broadly due to two factors. Firstly, there was a perception that LTBI incidence would be low in under-served populations*:* “*I think historically there aren’t a great number of cases from that group*” (#1); “…*it’s just not high on the priority, and because, I think, it’s small numbers*” (#6). Secondly, that in people diagnosed with LTBI, the rates of treatment completion would be low. It was notable that whilst some of this scepticism was based on previous personal experience, much of this sentiment seemed to be grounded in general perceptions about under-served populations at large.

“… *it was very labour intensive for very little reward really. I mean, you’re trying to get a population that’s doesn’t really want to engage with anything… … we weren’t very successful in treating very many of them*” (#11)

*“… in all honesty I think it’s a heck of a lot of work for little benefit”* (#11)

#### Targeting TB elimination

All participants were fundamentally positive about the opportunity to diagnose and treat latent TB in under-served populations: “*…it’s a good idea. You know, they’re a group at risk… why wouldn’t you?*” (#16). However, given the interview context in which we were referring to a nationally recommended service, it is unlikely that contrary views would have been expressed. Further exploration of participant support for LTBI testing led to discussions about TB elimination, and the role of LTBI management achieving this goal: “*obviously I can see the benefits of reducing the pool of latent TB infection in order to eventually reduce your burden of active TB*” (#11). Additionally, many went on to argue that aggressively identifying and treating latent disease may reduce the numbers presenting with active disease at a local level, potentially reducing their services future workload. The management of active TB in under-served populations, including directly observed treatment and often particularly labour-intensive contact tracing, was considered a real challenge, with participant accounts emphasising that TB reduction initiatives for this population should be prioritised: “*an active case in those populations is such a nightmare that just preventing one…”* (#4).

Participants recognised that to achieve national goals for TB elimination, the management of LTBI needed to be scaled up. This was considered particularly crucial for under-served populations, with the proportion of people with TB and more than one social risk factor increasing over the last few years [6]:

“*I do think that it's [LTBI management] worthwhile and I think if you're gonna eliminate TB, I think it's a strategy that [needs to] focus on the higher risk groups or under-served groups*” (#15)

The need for equitable care for those traditionally—and currently—under-served, was also noted as a concern:

“*we are making no difference in the populations who are most disproportionately affected by TB, and I think that’s a major problem*” (#9); “*they are as deserving as any other group we have who are likely to progress to TB and actually with more ramifications in terms of health*” (#5).

Notable is the reference to patient ‘deservedness’ in the quote above, potentially indicating a barrier relatively unexplored – the role of stigma in preventing the prioritisation of targeted care provision for marginalised populations.

## Discussion

This study explored the policy-practice mismatch for the management of LTBI in under-served populations, drawing on qualitative interviews with healthcare provider stakeholders. Results from thematic analysis are presented against two themes—service level challenges and low prioritisation – with participant identified enablers including integration with other services and alignment with TB elimination strategy.

Providing LTBI care to under-served populations was identified as challenging for several reasons. Diagnostic, therapeutic, and practical challenges make it difficult to accurately identify the appropriate subgroup for treatment, for whom the benefits will likely outweigh the risks, and for whom completion of treatment is feasible with the current model of care. Of these challenges, the most commonly cited concern was the ability to safely manage the risk of treatment related toxicity. This was partly ascribed to the recognised increased risk of hepatotoxicity or drug-drug interactions (e.g. rifampicin-methadone) in under-served populations [18] due to increased rates of alcohol and opiate use, but also to the perceived reduced ‘reliability’ of individuals in these groups.

We, as researchers, believe that clients should not be considered as “hard-to-reach”, with clinic non-attendance often due to a range of issues including stigma [19]. With this lens, we feel that whilst risks of treatment were perceived by participants as higher in under-served populations, the real issue is the inability of current care models to adequately mitigate against them. High completion rates of LTBI treatment have been successfully achieved in community-based approaches both in homeless [20] and incarcerated populations [21].

In line with this are the potential solutions cited by participants in this study, including integrating LTBI care with community-based healthcare settings such as opiate substitution therapy services, an approach with high participation rates in one study [22]. Re-designing services to this effect may improve the patient-service ‘connection’, potentially increasing prescriber confidence, and turn this challenge into an opportunity to provide excellent patient-centred care whilst up-skilling other healthcare cadres. An additional solution, not cited by participants, is the use of 3HP, a weekly directly observed regimen with evidence of safety and excellent completion rates in people experiencing homelessness [23, 24].

Ineffective commissioning and lack of funding were identified by participants as a barrier to provision of UK-based LTBI services. The lack of evidence on cost effectiveness was cited as rationale for lack of funding, but reportedly successful pilots did not seem to have moved this issue forward. It is vital that any pilots in the future generate adequate data to inform funding decisions, but this study suggests that low prioritisation may be a major barrier to this. This lack of prioritisation was related to clinician or system inertia, including lack of leadership from the TBCBs, was at least in part blamed on COVID-19. The pandemic has setback TB elimination goals by many years [25], but directing resources into LTBI is an important component to regaining lost ground in the fight against TB. In keeping with this, participants frequently alluded to switching the narrative to highlighting effective LTBI management as a requirement for the elimination of TB, with this focus improving drive from leadership and increasing investment to achieving adopted national and international goals for disease control. Even with such a change in priority, nationally commissioned work to answer cost-effectiveness questions, which allows regional adaptation to match local epidemiology, is necessary. These findings should be viewed in the context of LTBI management entering national and international guidance relatively recently, with some countries yet to incorporate recommendations for TB infection treatment.

Our findings are consistent with similar, previous studies on barriers to LTBI service provision, with low prioritisation having emerged has a key theme regarding LTBI management in Canada [26] and lack of resource, amongst other factors, cited in a UK study of primary healthcare professionals [27]. Previous research considering barriers to provision of other services to under-served populations have also found similar results: in Europe, lack of service co-ordination was cited as a barrier to mental health care provision for people experiencing homelessness [28], and a systematic review assessing homeless peoples’ experiences of healthcare found service level concerns including access to care to be a major theme [29].

It was notable that there were very few comments about potential practical solutions to service level challenges despite these topics being included in the topic guide. These include the use of rifapentine-based regimens, shown to improve outcomes in under-served populations [20, 24, 30], novel diagnostics such as c-TB, which may remove some practical challenges such as venepuncture [31], use of peer support / outreach workers [32, 33], and video-observed-treatment (VOT) [34, 35]. Workforce training to ensure optimal use of evidence-based strategies may help target some of these short-comings. Protocolising a cost-effective model of care could allow scale up in allied service provider settings – e.g. opiate substitution therapy services. Additionally, the use of novel bio-markers to assess risk of progression to active TB could improve population stratification.

To our knowledge this is the first study to look specifically at barriers to providing LTBI care to under-served populations. Strengths of this study include good recruitment, with participants with a range of healthcare backgrounds, and from a wide geographical area. A broad range of topics were covered, and data saturation was achieved. The use of inductive thematic analysis, allowing themes to emerge, also meant that results were data driven.

Limitations include the COVID-19 pandemic which has had a major impact on LTBI management in the UK, and our findings may not remain true in the future. Additionally, given the devolved nature of TB management in the UK, participants may not necessarily have represented their entire region, and we may have failed to capture the full extent of variation in practice.

## Conclusion

In the UK context, finding and treating LTBI in under-served populations is considered clinically and logistically challenging, of low public health value, and is an under-resourced and deprioritised component of TB control. Achieving the UK’s commitment to TB elimination goals will require accelerating efforts to scale-up both curative and preventive measures, including the management of LTBI. This will require targeted action and investment to strengthen the evidence base on integrated service delivery models, evaluate new treatment regimens and novel diagnostics, and allay workforce concerns. The current UK stance symbolises and potentially amplifies the extreme health inequities faced by populations at increased risk of TB in low incidence countries.

## Supplementary Information

**Additional file 1.** Interview guide.

## Data Availability

The datasets generated and analysed during the current study are not publicly available due to need to maintain participant anonymity, but are available from the corresponding author on reasonable request.

## Abbreviations

DOT: Directly observed therapy
ICB: Integrated care boards
LTBI: Latent tuberculosis infection
NICE: National Institute for Health and Care Excellence
TB: Tuberculosis
TBCB: Tuberculosis Control Board
UK: United Kingdom
WHO: World Health Organisation

## References

[1] World Health Organisation (2020). Consolidated guidelines on tuberculosis. Module 1: Prevention.

[2] Pareek M, Watson JP, Ormerod LP (2011). Screening of immigrants in the UK for imported latent tuberculosis: a multicentre cohort study and cost-effectiveness analysis. Lancet Infect Dis..

[3] National Institute for Health and Care Excellence. *Tuberculosis* [NICE Guideline 33). 2016. Available at: https://www.nice.org.uk/guidance/ng33. Accessed 23 Sep 2021. 31869029

[4] Public Health England. Collaborative TB Strategy for England 2015–2020: End of programme report. Public Health England, London. 2021. Available at: https://www.gov.uk/government/publications/collaborative-tuberculosis-strategy-for-england. Accessed 23 Sep 2021.

[5] Aldridge RW, Hayward AC, Hemming S (2018). High prevalence of latent tuberculosis and bloodborne virus infection in a homeless population. Thorax.

[6] Public Health England (2020). Tuberculosis in England, 2020 Report (presenting data to end of 2019).

[7] Deiss RG, Rodwell TC, Garfein RS. Tuberculosis and drug use: review and update. Clin Infect Dis. 2009;48(1):72–82. 10.1086/594126 PMC3110742 19046064

[8] Reichman LB, Felton CP, Edsall JR (1979). Drug dependence, a possible new risk factor for tuberculosis disease. Arch Intern Med.

[9] Yamin A, Bornstein E, Hensel R (2016). Predictors of latent tuberculosis infection treatment after introduction of a new regimen: a retrospective cohort study at an inner city clinic. Open Forum Infect Dis.

[10] Public Health England (2019). Tackling tuberculosis in under-served populations: a resource for TB control boards and their partners.

[11] Aldridge RW, Story A, Hwang SW (2018). Morbidity and mortality in homeless individuals, prisoners, sex workers, and individuals with substance use disorders in high-income countries: a systematic review and meta-analysis. Lancet.

[12] UK Health Security Agency (2021). TB action plan for England, 2021–2026.

[13] Spruijt I, Erkens C, Suurmond J (2019). Implementation of latent tuberculosis infection screening and treatment among newly arriving immigrants in the Netherlands: a mixed methods pilot evaluation. PLoS ONE.

[14] Faust L, Ruhwald M, Schumacher S (2020). How are high burden countries implementing policies and tools for latent tuberculosis infection? A survey of current practices and barriers. Health Sci Rep.

[15] Tong A, Sainsbury P, Craig J (2007). Consolidated criteria for reporting qualitative research (COREQ): a 32-item checklist for interviews and focus groups. Int J Qual Health Care.

[16] Braun V, Clarke V (2006). Using thematic analysis in psychology. Qual Res Psychol.

[17] Hamada Y, Cirillo DM, Matteelli A (2021). Tests for tuberculosis infection: landscape analysis. Eur Respir J.

[18] Shah M, Dorman SE (2021). Latent tuberculosis infection. N Engl J Med.

[19] Harris M (2020). Normalised pain and severe health care delay among people who inject drugs in London: Adapting cultural safety principles to promote care. Soc Sci Med.

[20] Nyamathi A, Salem BE, Shin SS (2021). Effect of a nurse-led community health worker intervention on latent tuberculosis medication completion among homeless adults. Nurs Res.

[21] Schmit KM, Lobato MN, Lang SG (2019). High completion rate for 12 weekly doses of isoniazid and rifapentine as treatment for latent mycobacterium tuberculosis infection in the federal bureau of prisons. J Public Health Manag Pract.

[22] Honarvar B, Lankarani KB, Odoomi N (2013). Pulmonary and latent tuberculosis screening in opiate drug users: an essential and neglected approach for harm-reduction facilities. J Addict Med.

[23] Sterling TR, Villarino ME, Borisov AS (2011). Three months of rifapentine and isoniazid for latent tuberculosis infection. N Engl J Med.

[24] Nwana N, Marks SM, Lan E (2019). Treatment of latent Mycobacterium tuberculosis infection with 12 once weekly directly-observed doses of isoniazid and rifapentine among persons experiencing homelessness. PLoS ONE.

[25] McQuaid CF, McCreesh N, Read JM (2020). The potential impact of COVID-19-related disruption on tuberculosis burden. Eur Respir J.

[26] Milinkovic DA, Birch S, Scott F (2019). Low prioritization of latent tuberculosis infection – a systemic barrier to tuberculosis control: a qualitative study in Ontario Canada. Int J Health Plan Manage.

[27] Atchison C, Zenner D, Barnett L (2015). Treating latent TB in primary care: a survey of enablers and barriers among UK general practitioners. BMC Infect Dis.

[28] Canavan R, Barry MM, Matanov A (2012). Service provision and barriers to care for homeless people with mental health problems across 14 European capital cities. BMC Health Serv Res.

[29] Omerov P, Craftman AG, Mattsson E (2020). Homeless persons’ experiences of health- and social care: a systematic integrative review. Health Soc Care Comm.

[30] Sandul AL, Nwana N, Hoilcombe JM (2017). High rate of treatment completion in program settings with 12-dose weekly isoniazid and rifapentine for latent mycobacterium tuberculosis infection. Clin Infect Dis.

[31] Hoff ST, Peter JG, Theron G (2016). Sensitivity of C-TB: a novel RD-1-specific skin test for the diagnosis of tuberculosis infection. Eur Respir J.

[32] Rinehart DJ, Stowell M, Collings A (2021). Increasing access to family planning services among women receiving medications for opioid use disorder: a pilot randomized trial examining a peer-led navigation intervention. J Subst Abuse Treat.

[33] Keats J, Micallef M, Grebely J (2015). Assessment and delivery of treatment for hepatitis C virus infection in an opioid substitution treatment clinic with integrated peer-based support in Newcastle Australia. Int J Drug Policy.

[34] Wong YJ, Ng KY, Lee SWH (2022). Digital health use in latent tuberculosis infection care: a systematic review. Int J Med Inform.

[35] Story A, Garber E, Aldridge RW, et al. Management and control of tuberculosis control in socially complex groups: a research programme including three RCTs. Southampton (UK): NIHR Journals Library; 2020. PMID: 33119243 33119243

