# Supplementary material for: “It’s too hard” – the management of latent TB in under-served populations in the UK: a qualitative study
Source: BMC Health Serv Res. 2022 Dec 1;22:1464. doi: 10.1186/s12913-022-08855-w (PMC9715280; doi:10.1186/s12913-022-08855-w)
Supplement: Supplementary file 1 — Additional file 1. Interview guide. [file 12913_2022_8855_MOESM1_ESM.docx]

# Survey of UK TB Control Boards on their practice and policy regarding latent TB in under-served populations: INTERVIEW GUIDE

The first part of the interview will document baseline information regarding the region being discussed including:

- Interviewees role
- Demographics for region: up-to-date TB epidemiology for active/latent disease
- Prevalence of USP: homeless, IVDU, incarcerated, vulnerable migrants (if known)
- Current practice for active case finding for active TB
- Current practice for new migrant LTBI screening including number screened, number diagnosed, number initiated on treatment and number completed treatment (if known)
- Current practice for other USP LTBI screening (using subsections)
  - People who inject drugs
  - People who are homeless
  - Sex workers
  - People involved with the justice system
  - Vulnerable migrants
- Diagnostic tool used IGRA vs TST (or other)
- Regimen choice for LTBI management, including any decision making tools for deciding which to use

The second part of the interview will explore barriers and facilitators to LTBI screening for USP (NB: not including new migrants) and also areas of innovation and solutions that have been identified to these challenges. This will initially be discussed using broad, open questions.

*“What do you think about latent TB screening in USP in your area?”*

“What do you see as the key barriers to screening USP for LTBI?”

*“How could LTBI screening be integrated with current TB active case finding? Or with other similar services?”*

*“How does HCV treatment work for USP in your area?”*

We will then discuss each part of the cascade of LTBI management.

“How do you screen everyone that is intended for screening?”

“How do you ensure all those tested get a result?”

“What is the pathway for referring a positive result to the appropriate service?”

“How is medical evaluation offered?”

“What is the process for decided on and recommending treatment?”

“Who initiates treatment and in what setting?”

“How are patients supported to ensure adherence and completion?”

Where appropriate we will break each part of the care cascade down using subsections for different groups with USP.

We will also aim to discuss some specifics of the regional TB programme and whether these practices are used for active TB, latent TB, or both.

- Use of peer support
- Management of methadone-rifampicin interaction
- Alcohol / isoniazid interaction
- Management of LTBI – HCV co-infection
- Use of DOT/VOT

*“Is there anything else you’d like to mention or discuss?”*
